# Hsc70 Contributes to Cancer Cell Survival by Preventing Rab1A Degradation under Stress Conditions

**DOI:** 10.1371/journal.pone.0096785

**Published:** 2014-05-06

**Authors:** Masako Tanaka, Saya Mun, Akihito Harada, Yasuyuki Ohkawa, Azusa Inagaki, Soichi Sano, Katsuyuki Takahashi, Yasukatsu Izumi, Mayuko Osada-Oka, Hideki Wanibuchi, Masayo Yamagata, Tokihito Yukimura, Katsuyuki Miura, Masayuki Shiota, Hiroshi Iwao

**Affiliations:** 1 Department of Pharmacology, Osaka City University Medical School, Osaka, Japan; 2 Department of Advanced Medical Initiatives, Faculty of Medical Sciences, Kyushu University, Fukuoka, Japan; 3 Department of Pathology, Osaka City University Medical School, Osaka, Japan; 4 Department of Cardiovascular Medicine, Osaka City University Medical School, Osaka, Japan; 5 Laboratory of Clinical Pharmacology, Faculty of Pharmacy, Osaka Ohtani University, Tondabayashi, Japan; 6 Applied Pharmacology and Therapeutics, Osaka City University Medical School, Osaka, Japan; Case Western Reserve University, United States of America

## Abstract

Heat shock cognate protein 70 (Hsc70) acts as a molecular chaperone for the maintenance of intracellular proteins, which allows cancer cells to survive under proteotoxic stress. We attempted to use Hsc70 to identify key molecules in cancer cell survival. Here, we performed mass-spectrometry-based proteomics analysis utilizing affinity purification with anti-Hsc70 antibodies; as a result, 83 differentially expressed proteins were identified under stress conditions. This result implies that there was a change in the proteins with which Hsc70 interacted in response to stress. Among the proteins identified under both serum-depleted and 5-fluorouracil-treated conditions, Rab1A was identified as an essential molecule for cancer cell survival. Hsc70 interacted with Rab1A in a chaperone-dependent manner. In addition, Hsc70 knockdown decreased the level of Rab1A and increased the level of its ubiquitination under stress conditions, suggesting that Hsc70 prevented the degradation of Rab1A denatured by stress exposure. We also found that Rab1A knockdown induced cell death by inhibition of autophagosome formation. Rab1A may therefore contribute to overcoming proteotoxic insults, which allows cancer cells to survive under stress conditions. Analysis of Hsc70 interactors provided insight into changes of intracellular status. We expect further study of the Hsc70 interactome to provide a more comprehensive understanding of cancer cell physiology.

## Introduction

The heat shock protein 70 family (Hsp70s) is a well-known group of molecular chaperones that support protein synthesis *in vivo*, assist in the folding of nascent polypeptides, and prevent the formation of aggregates by stopping non-specific interactions [1], [2]. In the presence of misfolded and denatured proteins, Hsp70s also facilitate their refolding or, in the case of irreparably impaired proteins, their removal by the protein degradation machinery of the cell [3]–[5]. Hsp70s are expressed at a high level in cancer cells and play a role in the maintenance of protein homeostasis, which promotes cancer cell growth and survival [6], [7]. Hsp70s are also known to be involved in tumor development, including metastasis, invasion, and drug resistance [8]–[11]; as such, several inhibitors targeting Hsp70s have been developed as anti-cancer agents [12]–[15].

Hsc70 is a constitutively expressed molecular chaperone that belongs to the Hsp70 family. It has some structural and functional similarities to Hsp72. However, Hsc70, which is not induced by heat shock or other stresses, maintains protein homeostasis under both normal and stress conditions, whereas stress-inducible Hsp72 is important for allowing cells to cope with acute stress. Hsc70 is reported to be involved in a multitude of housekeeping chaperoning functions, including the folding of nascent polypeptides, protein translocation across membranes, chaperone-mediated autophagy, the prevention of protein aggregation under stress conditions, and the disassembly of clathrin-coated vesicles [16]. Hence, Hsc70 knockout mice cannot be created due to the essential role of this protein in cell survival [17].

Cancer cells experience multiple microenvironmental stresses, such as hypoxia, nutrient deprivation, and exposure to chemotherapeutic agents [18]–[21]. In addition, malignant cells suffer from internal stresses, such as the accumulation of mutated and incorrectly folded proteins and the inappropriate activity of deregulated signaling pathways, which also threaten their survival [22]. Therefore, cancer cells need to overcome proteotoxic stress that arises by the intracellular accumulation of misfolded proteins. Similar to Hsp72, the expression of Hsc70 is higher in some cancer cell lines than in normal ones [23]. Although Hsc70 is overexpressed in cancer cells, little is known about how Hsc70 contributes to cancer cell survival compared with Hsp72. Under normal growth conditions, cytoplasmic Hsc70 can move in and out of the nucleus. However, it is concentrated in the nucleus when cells are exposed to stresses such as heat shock. This nuclear Hsc70 then relocates to the cytoplasm during recovery [24], [25]. Heat was also shown to induce rapid Hsc70 binding to insoluble cytoplasmic structures that are distinct from the cytoskeleton and internal cell membrane [26]. Hsc70 therefore acts rapidly in response to stress, but is constitutively expressed. There is also a change in the proteins with which Hsc70 interacts upon exposure to stress. Under proteotoxic conditions, the function of Hsc70 in assisting cotranslational folding is interrupted and this molecule instead facilitates the repair of misfolded proteins [27]. Therefore, Hsc70 client proteins are important for cellular homeostasis and are essential molecules in stress responses. Since Hsc70 is required for the folding of client proteins, its client proteins under stress conditions are potentially key molecules for the survival of cancer cells.

In this study, we performed mass-spectrometry-based proteomics analysis utilizing affinity purification with anti-Hsc70 antibodies for Hsc70 interactome profiling of the human colon cancer cell line HT29. By comparing the profiles of Hsc70 client proteins between normal and stress conditions, we identified molecules that are potentially critical for cancer cell survival.

## Materials and Methods

### Primary Antibodies and Chemicals

Antibodies against Rab1A, ubiquitin, Hsp72, and p62 were purchased from Santa Cruz Biotechnology (Santa Cruz, CA). Antibodies against all forms of poly (ADP-ribose) polymerase-1 (PARP-1) and cleaved forms of caspase-3 were purchased from Cell Signaling Technology (Beverly, MA). Antibodies against β-actin and LC3B antibody were purchased from Sigma (St. Louis, MO). Anti-Rab1B antibody was purchased from Abgen (San Diego, CA). HRP-conjugated secondary antibodies were purchased from GE Healthcare Bio-Science (Little Chalfont, Bucks, UK). Two different anti-Hsc70 antibodies used in affinity purification were produced in our laboratory [25], and anti-Hsc70 antibody used in the other experiments was purchased from Enzo (Farmingdale, NY). MG132 was purchased from Sigma (St. Louis, MO). 5-Fluorouracil (5-FU), and brefeldin A (BFA) were obtained from Wako (Osaka, Japan), diluted in dimethyl sulfoxide (DMSO), and stored at −20°C.

### Cell Culture and Experimental Treatments

The human colonic adenocarcinoma cell line HT29 was purchased from DS Pharma Biomedical (Osaka, Japan) and maintained in McCoy’s 5A medium (Invitrogen) supplemented with 10% fetal bovine serum (FBS), 100 U/ml penicillin, and 100 U/ml streptomycin in a humidified incubator with 5% CO_2_ at 37°C. Cells were passaged every seven days when approaching confluence. Cells were treated with 3.2 µM (IC_50_ at 48 h) 5-FU or without FBS (serum depletion) for six hours. For mass-spectrometry-based proteomics, 10 µM 5-FU was used. All treatments were performed at a final concentration of 0.1% DMSO.

### Immunoblotting and Immunoprecipitation

Cells were lysed in RIPA buffer, and proteins were isolated from cell lysates by immunoprecipitation as described previously [28]. For immunoblotting, proteins were separated on SDS-polyacrylamide gels under reducing conditions, followed by electrophoretic transfer to PVDF membranes as described previously [29]. The membranes were detected with LAS-4000 lumino-image analyzer system (GE Healthcare Bio-Science) using the enhanced chemiluminescence technique (Immobilon Western HRP Substrate; Millipore).

### Isolation and Proteomics Analysis of Hsc70 Client Proteins

For mass spectrometry analysis, cells were washed with PBS and fixed with 1% paraformaldehyde at room temperature for 20 min, followed by quenching with 125 mM glycine. Fixed cells were lysed in RIPA buffer consisting of 10 mM Tris-HCl, pH 7.4, 150 mM NaCl, 5 mM EDTA, 1% Triton X-100, 10% glycerol, 100 mM NaF, 1 mM phenylmethylsulfonyl fluoride, 0.2 mM DTT, and protease inhibitor cocktail. Cell lysates (1 mg of protein) were precleaned with inactivated NHS-Sepharose beads (GE Healthcare Bio-Science) for one hour at room temperature and immunoprecipitated with two kinds of in-house antibody specific for HSC70 immobilized on NHS-activated Sepharose at room temperature for two hours. The immunoprecipitates were then washed three times with RIPA buffer, and subjected to desalting and concentration by SDS-PAGE on a 5–20% polyacrylamide gradient gel (Wako), followed by staining with Quick-CBB (Wako). The gel was excised at slices of ∼1 mm thickness per lane. The gel pieces were destained in 25 mM NH_4_HCO_3_ and 50% acetonitrile, followed by additional destaining in 25 mM NH_4_HCO_3_, 30% acetonitrile, and reduction in 25 mM NH_4_HCO_3_ containing 10 mM DTT at 56°C for one hour. The reduced cysteine residues were subsequently alkylated in 55 mM iodoacetamide in 25 mM NH_4_HCO_3_ and the gel pieces were then washed in acetonitrile and dried. The dried gel pieces were treated with 10 µg/ml trypsin (Trypsin Gold; Promega, Madison, WI) in 50 mM NH_4_HCO_3_ on ice for 30 min, the excess was removed, and complete digestion was allowed to occur at 37°C for 18 h in 50 mM NH_4_HCO_3_. The samples were desalted using Zip Tip C18 (Millipore) in accordance with the manufacturer’s instructions. Trifluoroacetic acid was then added to a final concentration of 0.1% and used for nano-liquid chromatography electrospray ionization-tandem mass spectrometry (LC-ESI-MS/MS) analysis.

### Nanoelectrospray LC-MS/MS Analysis and Protein Identification

LC-MS/MS analyses were performed on a DiNa-AI nano LC system (KYA Technology, Tokyo, Japan) coupled to a QSTAR Elite hybrid mass spectrometer (AB Sciex, Concord, Ontario, Canada) through a NanoSpray ion source (AB Sciex). The details of this analysis are described elsewhere [30]. Data acquisition was performed using Analyst QS Software 2.0 (AB Sciex) in the positive-ion mode. Both sets of data were processed by ProteinPilot using the Paragon™ search algorithm (AB Sciex). MS/MS data were used as a search query in the NCBI database (RefSeq release 55, September 2012, ftp://ftp.hgc.jp/pub/mirror/ncbi/refseq/) using a *Homo sapiens* taxonomy filter. The minimum threshold for protein identification was set at a protein score of 0.47, corresponding to a confidence level greater than 66% and a false discovery rate of 1%.

### iTRAQ Labeling and Quantification of Protein Expression

The proteins from control or Rab1A-silenced cells were extracted as described for immunoblotting. Cell lysates were concentrated and the dissolution buffer (100 mM triethyl-ammonium bicarbonate, pH 8.0) was replaced with Microcon centrifugal filters with a 3 K nominal molecular weight limit ultrafiltration membrane, followed by digestion and labeling with 4-plex iTRAQ reagents in accordance with standard procedures [31]. The samples were labeled as follows: 114, control knockdown; and 115, Rab1A knockdown. Each sample contained 100 µg of protein. Protein concentrations were measured by BCA protein assay.

### RNA Interference

All siRNAs against human *Hsc70* (*HSPA8*), *Rab1A*, and *Ran*, and silencer negative control number 1 siRNA (Control) were obtained from Invitrogen. Lipofectamine RNAiMAX (Invitrogen) was used to reverse-transfect siRNAs into cells (final concentration, 30 nM or 10 nM) in accordance with the manufacturer’s instructions.

### MTS Assays

siRNA-transfected cells were seeded in 96-well plates at a density of 1×10^4^ or 1×10^5^ cells per well. After 48 h of transfection, the cells were cultured with serum-depleted medium or with 5-FU (3.2 µM) for 24 h. The viability was determined by assaying with Cell Counting Kit-8 (Dojindo, Kumamoto, Japan). Absorbance was measured at 450 nm with a Microplate Reader.

### Apoptosis Assay

siRNA-transfected cells were seeded in 24-well plates at a density of 5×10^4^ cells per well. After 48 h of transfection, the cells were subjected to serum depletion or 5-FU treatment for 24 h. Apoptotic cells were identified by nuclear staining with Hoechst 33258 after fixation with 0.5% glutaraldehyde for five minutes at room temperature. Coverslips were mounted with Fluorescence Mounting Medium (Dako, Glostrup, Denmark). Stained cells were visualized using a fluorescence microscope (Biozero; Keyence, Osaka, Japan) with a 20× dry objective.

### RNA Preparation and Real-time Reverse Transcriptase-polymerase Chain Reaction Analysis

Total RNA was isolated from Hsc70- or Rab1A-knockdown, or control knockdown cells using ISOGEN reagent (Nippon Gene, Tokyo, Japan). Reverse transcription was then performed using 1 µg of RNA and a ReverTra Ace qPCR RT Master Mix with gDNA Remover (TOYOBO, Osaka, Japan). Real-time PCR analysis was carried out using a 7500 Fast Real-Time PCR System (Applied Biosystems, Foster City, CA) with the LightCycler-FastStart DNA Master SYBR Green I kit (Roche, Penzberg, Germany). The RNA level was normalized using *GAPDH*. All data were analyzed by comparative C_T_ using 7500 Software ver. 2.0.1 (Applied Biosystems, Foster City, CA).

### IncuCyte Cell Growth Measurement Assay

Forty-eight hours after siRNA transfection, a total of 5×10^4^ HT29 cells were seeded in 24-well plates. Untransfected cells were treated with 5 µg/ml BFA or DMSO at the onset of measurement. The plate was incubated in an IncuCyte kinetic imaging system (Essen BioScience, Ann Arbor, MI) inside a cell culture incubator. Images were captured in four fields per well every three hours to monitor proliferation.

### Statistical Analysis

All data are presented as mean ± S.D. Comparisons among groups were made by one-way analysis of variance. Differences were considered statistically significant at *p*<0.05.

## Results

### Hsc70 is Critical for Cancer Cell Survival

**Figure 1:**
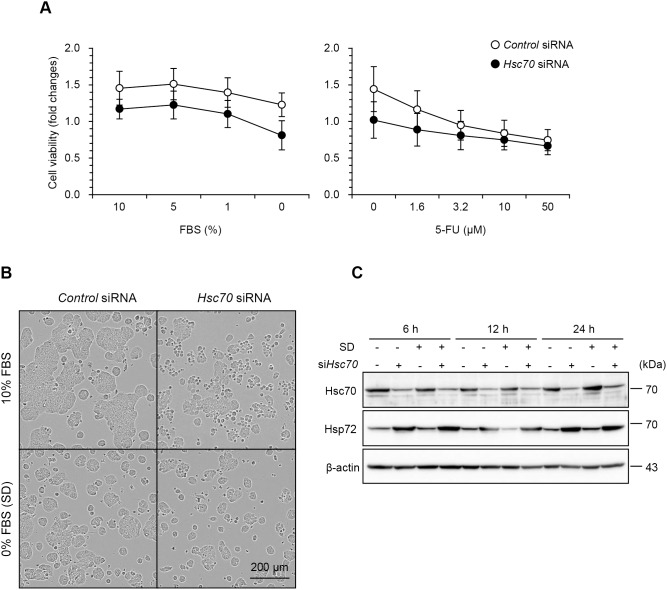
Hsc70 is critical for cancer cell survival. (A) Knockdown of Hsc70 decreased cancer cell viability. HT29 cells were transfected with siRNA for *Hsc70* or scramble control. At 48 h after transfection of siRNA, cells were subjected to serum starvation or were treated with 5-FU at the indicated concentration for 48 h. Cell viability was determined by MTS assay. Data points represent the mean ± S.D. (n = 5). (B) Effect of Hsc70 knockdown on cellular morphology. Hsc70 or control knockdown cells were treated the same as in *A*. Phase-contrast images of cells under serum-fed or serum-free conditions 24 h after treatments. Scale bar, 200 µm. (C) Hsc70 knockdown induced compensatory expression of Hsp72. At 48 h after transfection of siRNA, Hsc70 knockdown or control cells were subjected to serum depletion for 6, 12, or 24 h. After cell lysis, Hsc70 and Hsp72 were detected by immunoblotting. SD, serum depletion.

To investigate the contribution of Hsc70 to HT29 cell survival, we performed the MTS assay on Hsc70 knockdown cells (Fig. 1A). Compared with the control, Hsc70 knockdown decreased cell viability, which was independent of stress intensity. Although HT29 cells were particularly resistant to serum starvation, Hsc70 knockdown increased the sensitivity to serum starvation and led to cell death (Fig. 1B and Fig. S1). Despite the subsequent induction of Hsp72 as an adaptive stress response (Fig. 1C), Hsp72 did not prevent cell death induced by Hsc70 knockdown. Therefore, Hsc70 is critical for cell survival and Hsp72 could not completely compensate for its loss.

### Analyses of Hsc70 Interactors Identified in Serum-depleted and 5-FU-treated HT29 Cells

**Figure 2:**
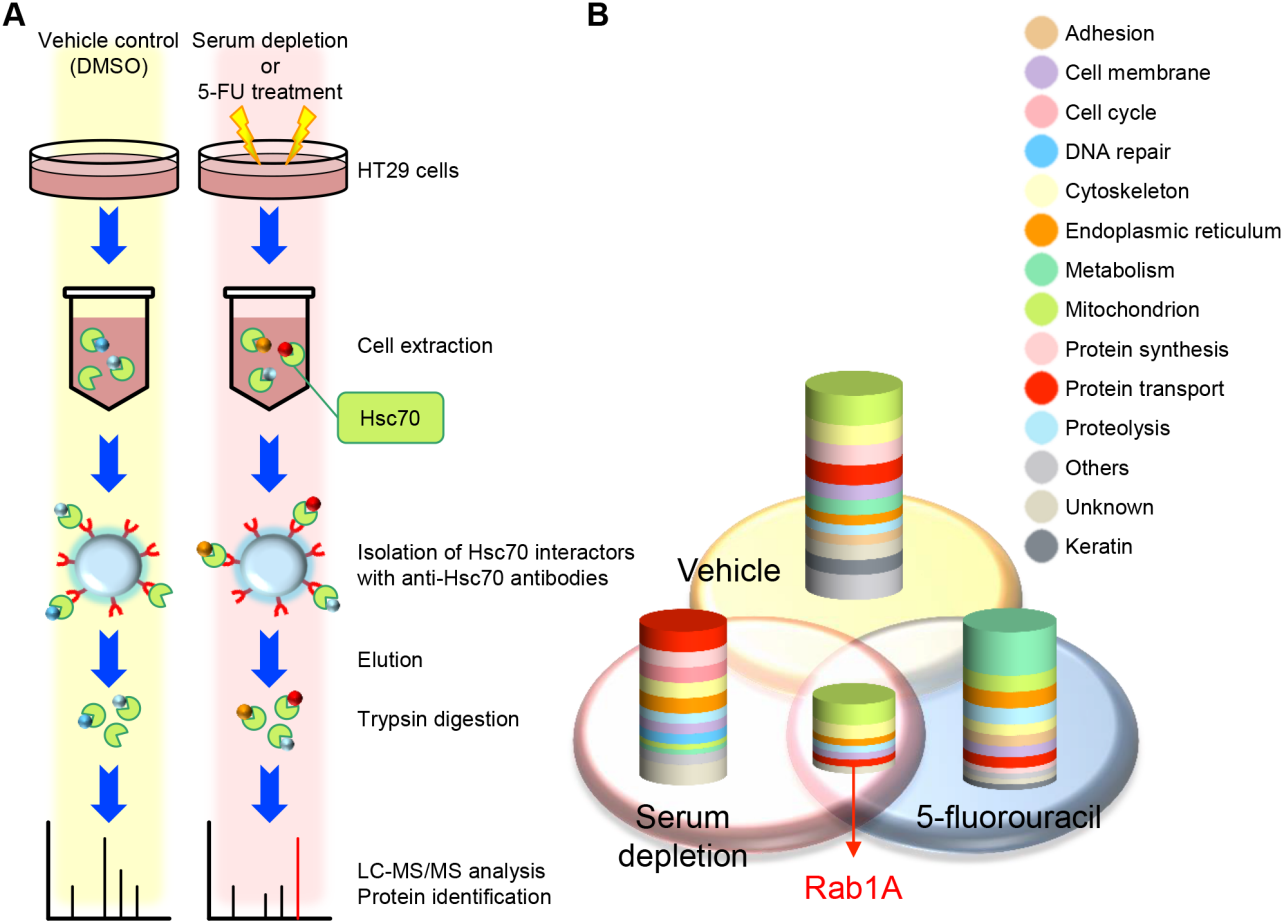
Hsc70 interactors identified in serum-depleted and 5-FU-treated HT29 cells. (A) A schematic view of the identification of Hsc70 interactors. Cells were exposed to serum depletion (SD), 5-FU, or DMSO for 6 h. Hsc70 interactors were isolated with anti-Hsc70 antibodies from the cell extract, and were identified through subsequent analysis by liquid chromatography-tandem mass spectrometry (LC-MS/MS). (B) Identification and functional classification of Hsc70 interactors that were associated with changes in stress response. A Venn diagram depicting Hsc70 interactors that were identified at >95% confidence score (1.3 ProtScore) using ProteinPilot 2.0 software. Column graphs indicate the number and functionality of identified proteins.

As a variety of stresses induce the intracellular accumulation of misfolded and denatured proteins, Hsc70 binds to and refolds these damaged proteins. This function of Hsc70 allows cells to survive under proteotoxic stresses. Thus, we attempted to analyze Hsc70 interactors under stress conditions in order to identify key molecules for cancer cell survival. A flowchart of the identification of Hsc70 interactors is shown in Fig. 2A. HT29 cells were exposed to serum depletion or 5-FU, and also treated with DMSO as a vehicle control for 6 h. Profiling of Hsc70 interactors in cancer cells was carried out by isolation using Hsc70 affinity beads, followed by mass-spectrometry-based identification. A total of 124 unique proteins were identified at >95% confidence (Fig. 2B and Table S1). Of these, 41 proteins unique to the vehicle control were involved in normal homeostatic processes. Gene Ontology (GO) analysis revealed that these proteins were involved in mitochondrial energy production, the cytoskeleton, and protein synthesis. Among the remaining proteins, 30 proteins unique to serum depletion were particularly involved in protein transport, protein synthesis, and the cell cycle, whereas 29 proteins unique to 5-FU treatment were commonly associated with glucose metabolism. These results indicate that there was a change in the proteins with which Hsc70 interacted in response to different types of stress. Therefore, the Hsc70 interactome reflects stimulus-dependent changes in the responses to stress and could be used to monitor the physiological changes of cells. Our analysis also identified a limited number of proteins that were common in both stress conditions, which were involved in mitochondria, the cytoskeleton, and protein transport. Of these, we focused on Rab1A in the Ras superfamily, which is expressed at a high level in cancer cells. This molecule regulates key cellular processes such as signal transduction, cell proliferation, and vesicle transport (Fig. 2B and Table S2).

### Rab1A Knockdown Exacerbated Stress Damage by Serum Depletion and 5-FU Treatment

**Figure 3:**
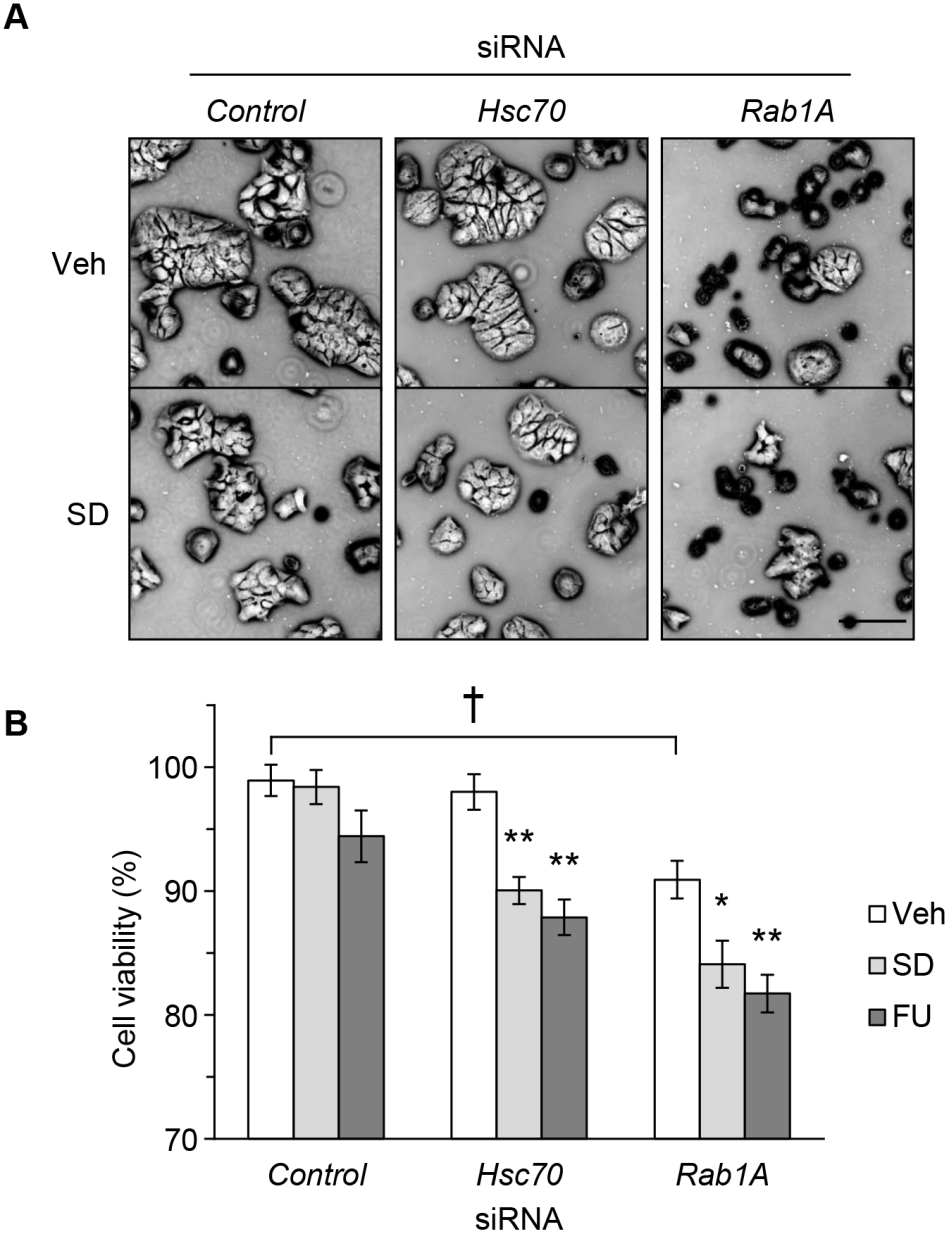
Rab1A knockdown exacerbated stress damage due to serum depletion and 5-FU treatment. HT29 cells transfected with *Hsc70*, *Rab1A*, or *control* siRNA were subjected to serum depletion, 5-FU, or vehicle treatment for 24 h. (A) Effects of suppression of Hsc70 and its interactors on cellular morphology. Representative phase-contrast images of cells. Scale bar, 100 µm. (B) Rab1A knockdown decreased cell viability. Cell viability was determined by MTS assay. Asterisks indicate statistical significance. **, *p*<0.01, *, *p*<0.05 vs. vehicle; ^†^, *p*<0.05 vs. control knockdown by two-way ANOVA followed by Tukey-Kramer post hoc test; values are the means ± S.D. (*n* = 7). Veh, vehicle. SD, serum depletion. FU, 5-fluorouracil.

To extend our proteomics findings, we next evaluated the contribution of Rab1A to cancer cell survival. Compared with control transfection, Hsc70 knockdown reduced colony size, without any alteration of cellular morphology (Fig. 3A). However, Rab1A knockdown caused attenuation of cell-cell adhesion and markedly decreased the cell number, which was enhanced by serum depletion. To quantify the effects of Rab1A knockdown, we performed the MTS assay (Fig. 3B). Cells were seeded at 1×10^5^ per well in 96-well plates in order to eliminate the influence of cell proliferation. Both serum depletion and 5-FU treatment had little effect on the viability of control cells, indicating that these treatments only constituted mild stress for the cells. Compared with control cells, Hsc70 knockdown significantly decreased cell viability (*p*<0.01) despite the mild stress exposure. This shows that Hsc70 knockdown induced cell death under stress conditions, as also indicated in Fig. 1A and B. However, Rab1A knockdown significantly decreased cell viability (*p*<0.05) without stress exposure, further indicating that Rab1A knockdown alone induced cell death under normal conditions. Furthermore, despite the mild nature of these stresses, serum depletion and 5-FU treatment further decreased the viability of Rab1A knockdown cells. These results indicate that Rab1A contributes to cell survival and is very important under stress conditions that are beyond the ability of Hsc70 to ensure cell survival.

### Hsc70 Prevented Rab1A Degradation

**Figure 4:**
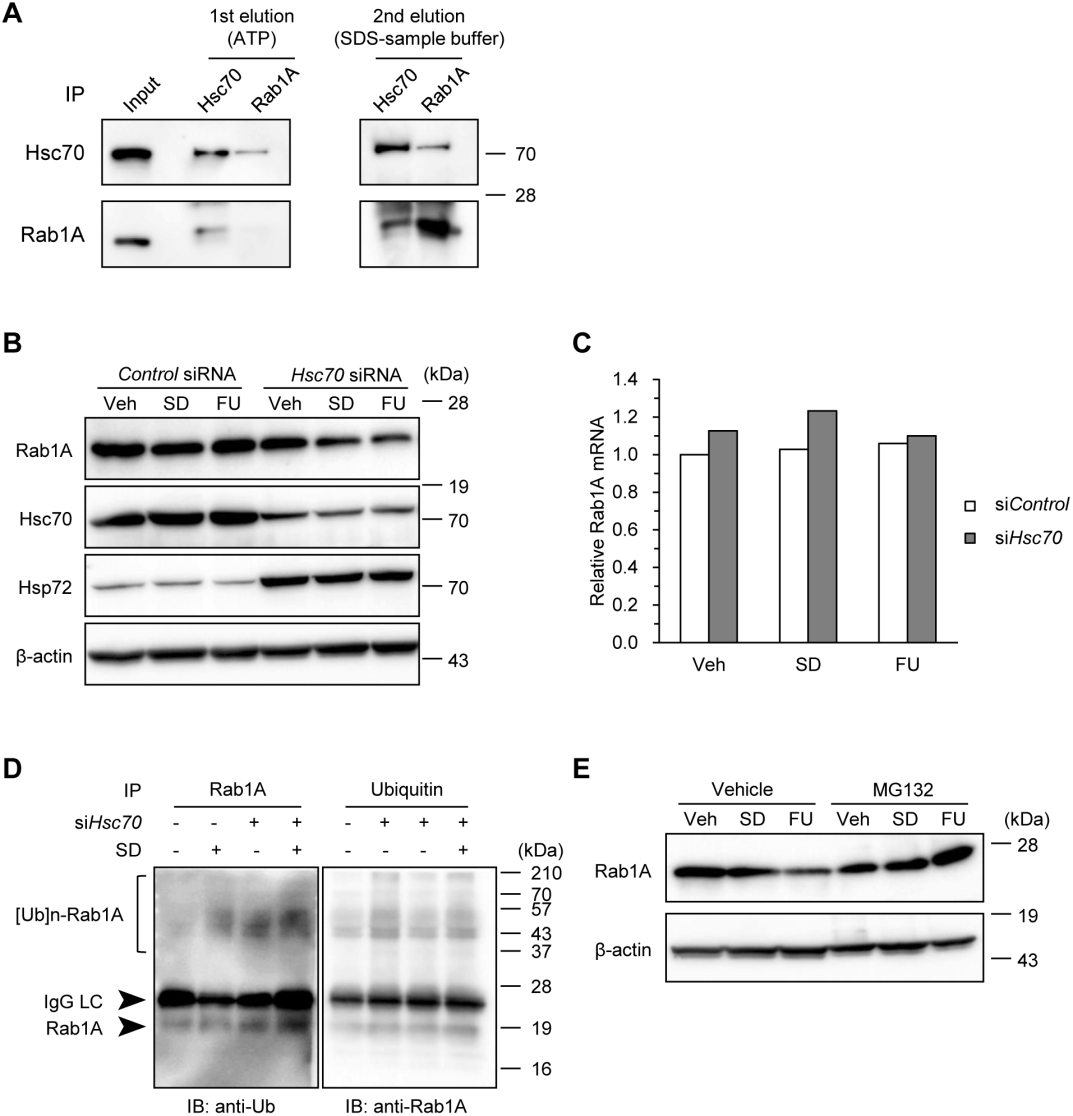
Hsc70 prevented Rab1A degradation. (A) Hsc70-Rab1A interaction through chaperone activity. HT29 cells were subjected to serum depletion for 24 h, and then lysed. To verify the interaction between Hsc70 and Rab1A in a chaperone-dependent manner, anti-Hsc70 or anti-Rab1A immunoprecipitant was eluted with ATP, followed by elution with SDS sample buffer and immunoblotting in order to confirm the bait proteins. (B) Hsc70 knockdown decreased the level of Rab1A protein under stress conditions. HT29 cells transfected with *Hsc70* or *control* siRNA were subjected to serum depletion, 5-FU, or vehicle treatment for 24 h. Immunoblotting for endogenous Rab1A and Hsc70 proteins. β-actin was used as a loading control. (C) Hsc70 knockdown did not decrease *Rab1A* mRNA level. After knockdown of Hsc70 and in the control, *Rab1A* mRNA levels were determined by qPCR at 48 h post-transfection. (D) Hsc70 knockdown promoted the ubiquitination of Rab1A. After Hsc70 knockdown or control cells were lysed, immunoprecipitation (IP) with anti-Rab1A or anti-ubiquitin antibodies was performed, followed by immunoblotting with anti-ubiquitin or anti-Rab1A antibodies. (E) MG132 treatment inhibited Rab1A degradation. Hsc70 knockdown cells were subjected to serum depletion or 5-FU treatment, and then MG132 (10 µM) or vehicle was added for the last 8 h before sampling. SD, serum depletion. FU, 5-fluorouracil. Ub, ubiquitin, IgG LC, immunoglobulin light chain. Data (except in C) are representative of at least two separate experiments yielding similar results.

To investigate the effects of Hsc70 on Rab1A under stress conditions, we focused on the interaction of these two molecules. We verified the physical interaction of endogenous Hsc70 and Rab1A by co-immunoprecipitation assays with commercial antibodies (Fig. 4A). This confirmed the results of the mass spectrometric analysis that Hsc70 interacted with Rab1A under serum-depleted conditions. We further confirmed that Hsc70 interacted with Rab1A in a chaperone-dependent manner because Hsc70 and Rab1A were disassociated from each other by ATP. We also found that Hsc70 formed homodimers, but Rab1A did not. Secondary elution in SDS sample buffer for the remaining proteins bound to agarose beads was performed to detect the bait proteins because ATP does not interfere with antigen-antibody interaction. Next, we evaluated Rab1A level when Hsc70 was suppressed; it showed a decrease under stress conditions despite a compensatory increase of Hsp72 level (Fig. 4B). However, Hsc70 knockdown had no effect on Rab1A level under the control conditions. This downregulation of Rab1A under stress conditions was observed at the protein level, but not at the mRNA level (Fig. 4C). Hsc70 was therefore shown not to regulate Rab1A synthesis, at least. We next found that Hsc70 suppression increased the level of ubiquitinated Rab1A under serum-depleted conditions (Fig. 4D). Furthermore, Rab1A degradation that was induced by Hsc70 knockdown under stress conditions was inhibited by the proteasome inhibitor MG132 (Fig. 4E). Collectively, these results suggest that Hsc70 prevents the degradation of Rab1A that has been misfolded under stress conditions.

### Rab1A Knockdown-induced Cell Death is not from Apoptosis

**Figure 5:**
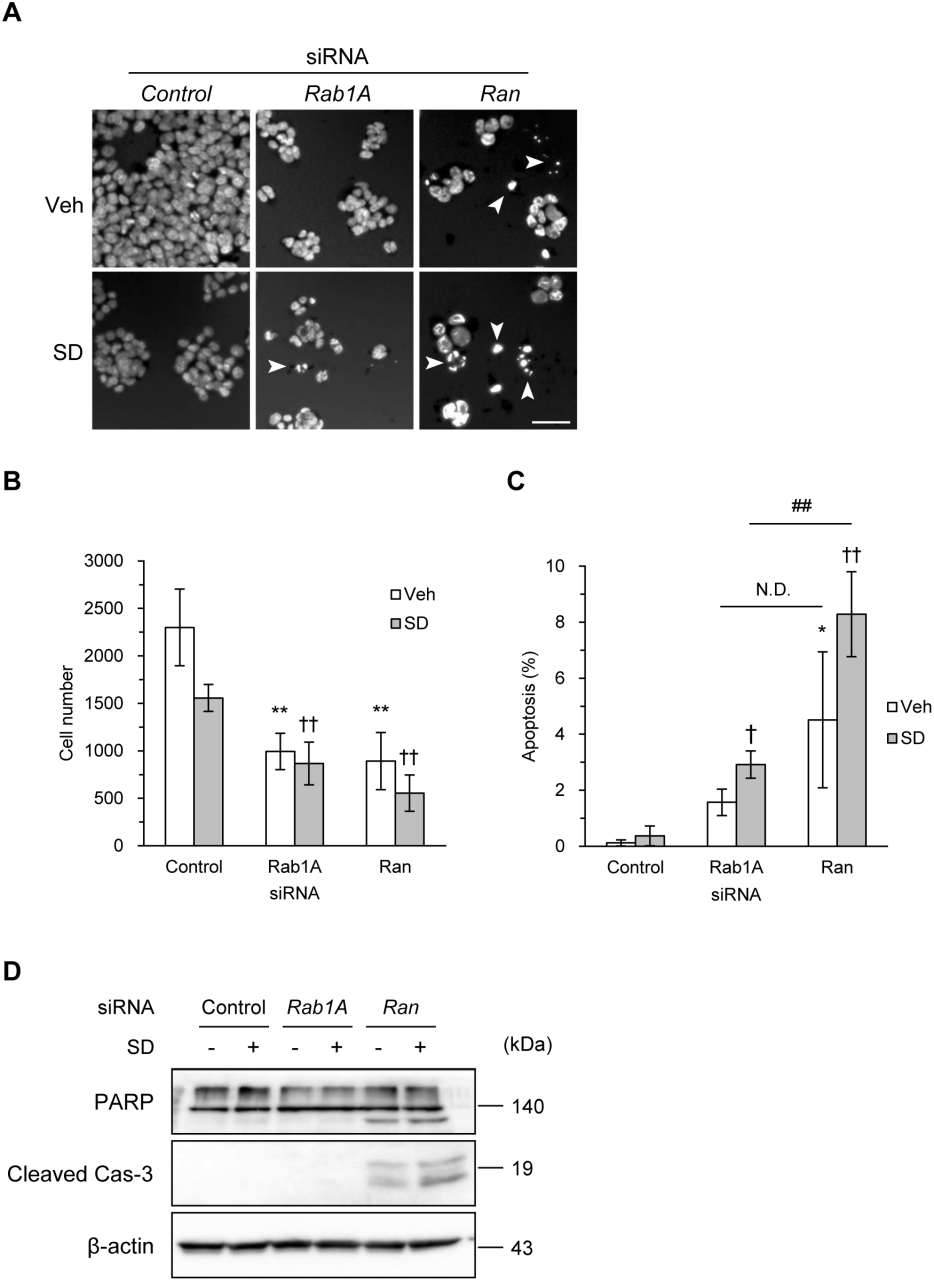
Rab1A knockdown induced cell death not including apoptosis. HT29 cells transfected with *Hsc70*, *Rab1A*, *Ran*, or *control* siRNA were subjected to serum depletion, 5-FU, or vehicle treatment for 24 h. (A) Rab1A knockdown had little effect on the induction of apoptosis. The induction of apoptosis was analyzed by staining with Hoechst 33258. White arrowheads indicate apoptotic nuclei with condensed chromatin. Scale bar, 50 µm. (B, C) The decrease in cell number by Rab1A knockdown was not due to apoptosis. Numbers of total cells and apoptotic cells were quantified by counting Hoechst-stained cells and cells with nuclear condensation in (A), respectively. *, *p*<0.05, **, *p*<0.01 vs. control/veh; ^†^, *p*<0.05, ^††^, *p*<0.01 vs. control/SD; ^##^, *p*<0.01 vs. Rab1A/SD by two-way ANOVA followed by Bonferroni/Dunn post hoc test; values are the means ± S.D. (*n* = 3). (D) Rab1A suppression did not induce apoptosis. Apoptosis was determined by the cleavages of PARP-1 and caspase-3, detected by immunoblotting. β-actin was used as a loading control. Veh, vehicle. SD, serum depletion. FU, 5-fluorouracil. Immunoblotting data are representative of at least three separate experiments yielding similar results.

To investigate whether the absence of Rab1A induces apoptosis, we examined the presence of apoptotic cells by nuclear staining with Hoechst 33258. Ran, a member of the Ras superfamily, interacted with Hsc70 under all conditions according to our proteomics analysis (Table S2). Ran knockdown was performed as a positive control for apoptosis. Ran knockdown cells exhibited apoptotic nuclear fragmentation independently of serum depletion, whereas Rab1A knockdown cells exhibited only slight apoptosis when exposed to serum depletion (Fig. 5A). Upon counting the Hoechst-stained cells and those with nuclear fragmentation, Rab1A knockdown was found to decrease the cell number to the same extent as Ran knockdown (Fig. 5B). However, apoptosis was markedly induced by Ran knockdown but not by Rab1A knockdown (Fig. 5C). Because we also did not detect typical PARP-1 and caspase-3 apoptotic signatures upon Rab1A knockdown (Fig. 5D), cell death exacerbated by Rab1A knockdown was shown not to be responsible for the apoptosis. Thus, these results suggest that Rab1A knockdown-induced cell death is not from apoptosis.

### Quantitative Differential Proteomics in Rab1A Knockdown Cells

**Figure 6:**
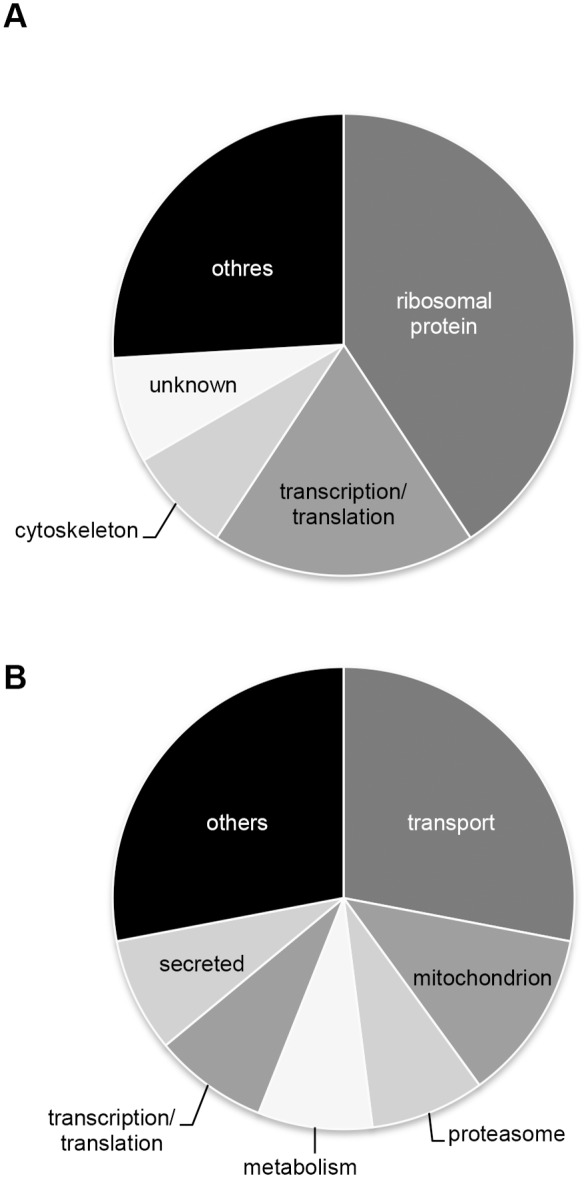
Functional annotation of upregulated or downregulated proteins in Rab1A knockdown cells. HT29 cells were transfected with siRNA for *Rab1A* or scramble control. Protein identification in Rab1A knockdown cells was performed through quantitative proteomics by stable isotope labeling, using iTRAQ. (A) Upregulated proteins with iTRAQ ratio ≥1.2. (B) Downregulated proteins with iTRAQ ratio <0.8.

To examine why the absence of Rab1A induced cell death not including apoptosis, we performed quantitative differential proteomics analysis of Rab1A knockdown cells based on the iTRAQ technique. The iTRAQ-labeled proteins that had been extracted from Rab1A knockdown cells were analyzed and compared with the proteome of control cells. As a result, 25 proteins with a change of expression of ≥1.2-fold were considered to be upregulated, whereas 27 proteins with a change <0.8-fold were downregulated (Table S3). To evaluate the functional differences between these two subsets of proteins, we performed GO analysis (Fig. 6A and B). The upregulated proteins were classified into the categories of protein transport, mitochondria, and proteasome, whereas classification under the terms ribosome and transcription/translation was particularly common among the downregulated proteins. These results suggested that Rab1A knockdown increased the levels of other proteins involved in endoplasmic reticulum (ER)-Golgi trafficking, but did not lead to compensation of Rab1A function. The dysfunction of Rab1A facilitated protein degradation and stopped protein synthesis, indicating that Rab1A knockdown in cells induced the accumulation of misfolded proteins. Hence, Rab1A knockdown cells may be exposed to proteotoxic stress, which subsequently induces cell death.

### Rab1A-knockdown-induced Cell Death was Caused by Inhibition of Autophagy but not ER-Golgi Traffic

**Figure 7:**
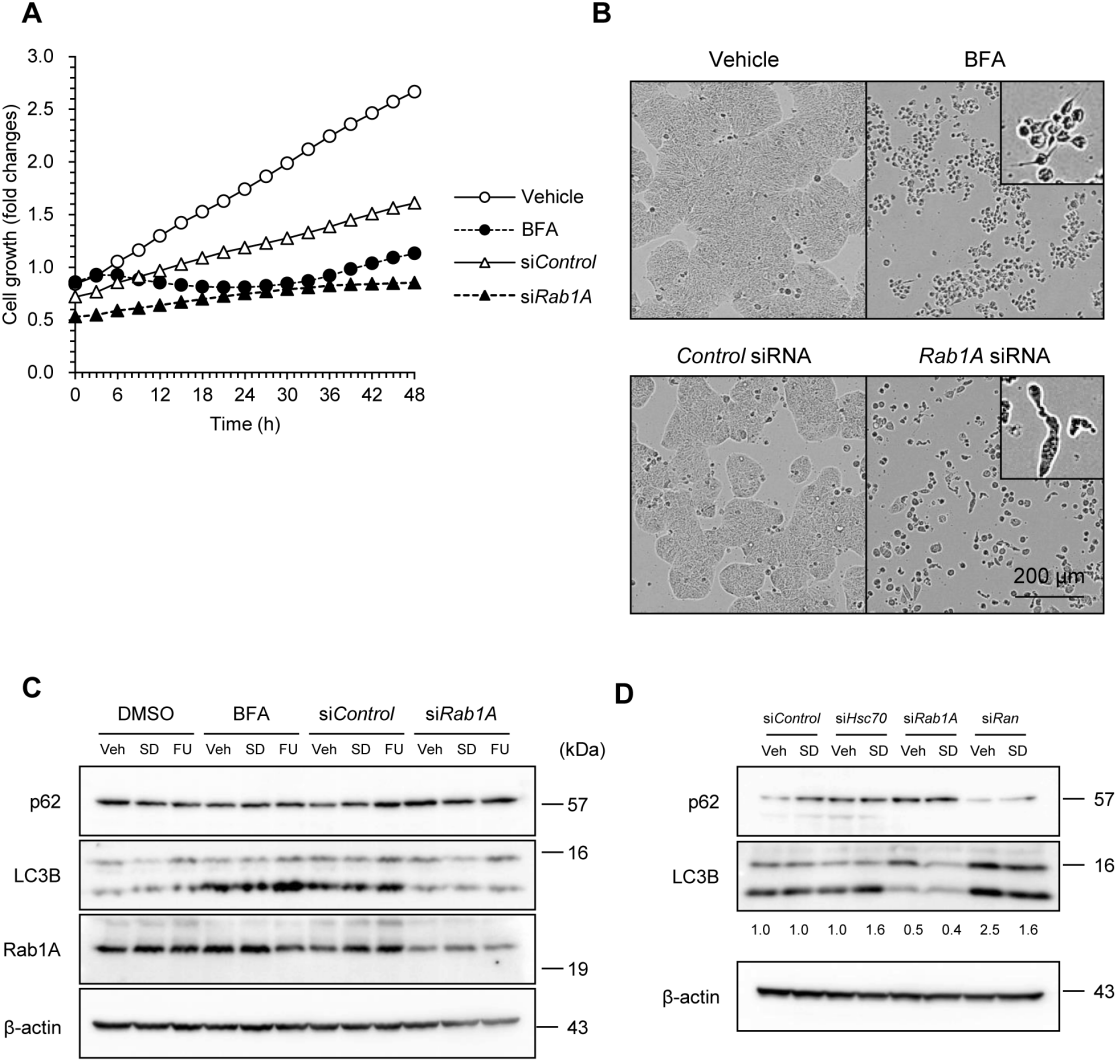
Rab1A-knockdown-induced cell death was caused by inhibition of autophagy but not ER-Golgi traffic. (A) BFA induced cell death differently from Rab1A knockdown. HT29 cells were transfected with *Hsc70* or *control* siRNA and untransfected cells were treated with 5 µg/ml BFA or DMSO at the onset of IncuCyte cell growth assay. Images were captured to monitor proliferation by IncuCyte inside a cell culture incubator. (B) Phase-contrast images at 48 h after the onset of measurement. Scale bar, 200 µm. (C) Inhibition of autophagy was not caused by the interruption of ER-Golgi traffic. Rab1A knockdown or control cells were subjected to serum depletion, 5-FU, or vehicle treatment for 24 h. Untransfected cells were treated with 5 µg/mL BFA for 6 h, and immunoblotted with anti-p62, LC3B, or Rab1A. β-actin was used as a loading control. (D) Hsc70 knockdown allowed autophagosome formation. HT29 cells transfected with *Hsc70*, *Rab1A*, *Ran*, or *control* siRNA were subjected to serum depletion or vehicle treatment for 24 h, and immunoblotted. Immunoblotting data are representative of at least two separate experiments yielding similar results.

Since Rab1A is involved in ER-Golgi traffic, we next examined whether interruption of this traffic induces cell death. Although treatment with BFA, an inhibitor of ER-Golgi traffic, induced cell death, the morphology of dying cells was vastly different from that upon Rab1A knockdown (Fig. 7A and B). Because BFA is also known as an ER stress inducer, BFA treatment is predicted to cause the intracellular accumulation of misfolded and denatured proteins. Therefore, we next investigated the induction of autophagy. As expected, BFA treatment induced LC3B–II, whereas Rab1A knockdown did not. Rab1A knockdown furthermore led to the accumulation of p62, which can bind LC3, thus serving as a selective substrate of autophagy (Fig. 7C). When Rab1A was suppressed, the mRNA level of Rab1B increased two-fold, whereas its protein level tended to increase slightly (Fig. S2A and B). Rab1A, therefore, may have a unique unknown function that Rab1B lacks or the total levels of Rab1A and Rab1B may decrease below the threshold necessary for cell survival when Rab1A was suppressed. Furthermore, since Hsc70 knockdown induced LC3B–II that allowed LC3B to become associated with autophagic vesicles, it did not affect the formation of autophagosomes, in contrast to Rab1A knockdown (Fig. 7D). Taking these findings together, the absence of Rab1A inhibits the formation of autophagosomes, leading to cell death.

## Discussion

The purpose of this study was to identify molecules that are critical to cancer cell survival, by focusing on the chaperone function of Hsc70, which assists in folding/refolding [16]. Our proteomics screening revealed that Hsc70 interactors showed stress-dependent changes, thereby providing the distinct cellular physiology corresponding to stress in HT29 cells. Although there were a few Hsc70 interactors common to the two different stresses, we identified Rab1A as contributing to cancer cell survival under stress conditions. Rab1A suppression decreased cell-cell adhesion and induced cell death. We also found that the absence of Rab1A inhibited not only ER-Golgi traffic but also the induction of autophagy, leading to the exacerbation of proteotoxic insults. Therefore, Hsc70 prevented Rab1A degradation under stress conditions, suggesting that it contributes to eliminate proteotoxic damage through the prevention of Rab1A degradation.

Stress-inducible Hsp72 is overexpressed in cancer cells. Several researchers have proposed that malignant cells are reliant on stress proteins, including Hsp72, for their survival [11], [13], [32]. However, it is largely unknown how Hsc70 contributes to cancer cell survival despite the well-known finding of its overexpression in malignant cells [16]. Since cancer cells experience multiple microenvironmental and intracellular stresses, they suffer from proteotoxic insults that threaten their survival [22], [33]. We previously reported that Hsc70 is translocated into the nucleus in response to oxidative stress [25]. Moreover, there is a change in the proteins with which Hsc70 binds in response to the accumulation of misfolded proteins, leading to pausing of the early elongation of nascent peptides and the rapid suppression of protein synthesis [27]. Therefore, the identification of Hsc70 interactors under stress conditions can clarify how Hsc70 contributes to cancer cell survival. In an attempt to elucidate the mechanism behind the stress response of cancer cells, this study was designed to explore the alteration of Hsc70 interactors in response to stress. On the basis of our analysis of Hsc70 interactors, we identified Rab1A as being involved in the viability of HT29 cells. Hsc70 interacted with Rab1A in a chaperone-dependent manner. Although Rab1A interacted with Hsc70 under stress conditions, there was no alteration in the protein expression of Rab1A in response to the stresses of serum depletion and 5-FU treatment. Most previous studies only enabled the identification of stress-inducible proteins, when searching for stress response proteins. The Hsc70 interactome, however, revealed that Rab1A was required for proteotoxic insults, but was constitutively expressed. The level of Rab1A was decreased by the knockdown of Hsc70. Hsc70 knockdown also enhanced the ubiquitination of Rab1A, suggesting that Hsc70 prevented the degradation of Rab1A that had been denatured by stress damage. Indeed, Rab1A knockdown decreased cell viability to the same extent as Hsc70 knockdown in combination with additional stresses. Although Hsc70 knockdown induced a compensatory increase of Hsp72, Hsp72 did not affect the degradation of Rab1A. Therefore, we revealed that Hsc70 has properties related to cancer cell survival that differ from those of Hsp72.

Rab1A knockdown induced cell death not including apoptosis, whereas Ran knockdown induced apoptotic cell death. Ran, which, like Rab1A, is a member of the small GTPases, has roles in nuclear transport, mitotic spindle assembly, and nuclear envelope assembly [34], [35]. Therefore, the absence of Ran rapidly induces apoptosis [36], [37]. Our proteomic data revealed that Hsc70 constantly interacted with Ran independently of stress. Since Hsc70 affects the GTPase cycle of Ran [38], Hsc70 modulates nucleocytoplasmic transport in concert with Ran. However, Hsc70 was shown to interact with Rab1A under stress conditions in order to prevent Rab1A degradation. On the basis of the results of our iTRAQ analysis, Rab1A knockdown induced proteotoxic stress similar to that due to Hsc70 knockdown. Therefore, we considered that the absence of Rab1A induced the misfolding and denaturing of proteins and disrupted intracellular regulation, which did not induce apoptosis. In addition, Rab1A knockdown inhibited the induction of LC3B–II, thereby inhibiting autophagosome formation [39]. Although Zoppino et al. stated that Rab1B is also involved in autophagosome formation [40], the compensatory increase of Rab1B that was induced by Rab1A knockdown was insufficient for autophagosome formation. These results indicate that the formation of autophagosomes in HT29 cells depends on Rab1A, or that the induced level of Rab1B is insufficient for autophagosome formation. Rab1A is localized in the ER-Golgi intermediate compartment and functions in the targeting and fusion of transport vesicles with their selective acceptor membrane [41]. However, BFA, an inhibitor of ER-Golgi traffic, did not affect autophagosome formation. Taking these findings together, we considered that cell death induced by Rab1A knockdown was primarily caused by the inhibition of autophagosome formation, but not ER-Golgi traffic. Recent evidence suggests that autophagy provides a protective function to limit tumor necrosis and inflammation, and to mitigate genome damage in tumor cells in response to metabolic stress [42]. In addition, ectopic expression of miR-502, which regulates Rab1B suppression, inhibits autophagy leading to colon cancer cell growth [43]. Therefore, Rab1 is overexpressed in cancer cells [44] because it may contribute to autophagy progression. Hsc70 is known to play an essential role in chaperone-mediated autophagy, which is responsible for the selective degradation of intracellular proteolysis [45]. However, Hsc70 is also involved in autophagosome formation through the prevention of Rab1A degradation.

Since Hsc70 plays a key role in cellular protein homeostasis, we utilized its chaperone function in our proteomics analysis in order to understand cancer cell physiology. The proteomics analysis of Hsc70 interactors identified Rab1A, which was shown to be required for cancer cell survival under stress conditions. The analysis of Hsc70 client proteins should provide a more comprehensive understanding of changes in the intracellular state at the protein level. We expect that the combination of our functional proteomics profiling and genome-wide mRNA profiling will be helpful to survey the full extent of physiological changes in cells through the monitoring of protein transitions.

## Supporting Information

Figure S1 **Hsc70 is critical for HT29 cell survival.** (A) At 48 h after transfection of siRNA, cells were subjected to serum depletion (0% FBS) or not (10% FBS) and cell growth was monitored using IncuCyte. Images were captured every 3 h, followed by quantification of cell area in these images. (B) At 48 h after transfection, cells were subjected to serum depletion (0% FBS) or not (10% FBS) for 24 h. Both attached and detached (floating) cells were collected for trypan blue exclusion assay. Cell viability was assessed by counting trypan blue-excluding cells; values are the means ± S.D. (n = 4).
(TIF)

Figure S2 **The effect of Rab1A knockdown on Rab1B expression.** (A) Rab1A knockdown or control cells were subjected to serum depletion, 5-FU, or vehicle treatment for 24 h. Untransfected cells were treated with 5 µg/mL BFA for 6 h, and immunoblotted with Rab1A or Rab1B. β-actin was used as a loading control. (B) After knockdown of Rab1A, *Rab1B* mRNA levels were determined by qPCR at 48 h post-transfection.
(TIF)

Table S1 Raw data list of the Hsc70 interactome. (A–C) This table includes all identified proteins with >47% confidence. These data constitute the unprocessed protein data output file of ProteinPilot. (D) This table contains the identified proteins of the Hsc70 interactome with a ProteinPilot unused score above 1.3, which is equivalent to a protein confidence level greater than 95%, and corresponds to Fig. 2B. Blue filled cells indicate the detected conditions.
(XLS)

Table S2 The raw data of Rab1A and Ran peptides. This table contains the corresponding peptides of Rab1A and Ran in Table S1. These data constitute the unprocessed peptide data output file of ProteinPilot.
(XLS)

Table S3 iTRAQ proteomic data of Rab1A or control knockdown cells. This table includes all identified proteins with >47% confidence. These data constitute the unprocessed protein data output file of ProteinPilot. The samples were labeled as follows: 114, control knockdown; and 115, Rab1A knockdown. Red shaded rows indicate upregulated proteins with iTRAQ ratio ≥1.2, whereas blue shaded rows indicate downregulated proteins with iTRAQ ratio <0.8.
(XLS)
