# Supplementary figures and images for: Hsc70 Contributes to Cancer Cell Survival by Preventing Rab1A Degradation under Stress Conditions
Source: PLoS One. 2014 May 6;9(5):e96785. doi: 10.1371/journal.pone.0096785 (PMC4011886; doi:10.1371/journal.pone.0096785)

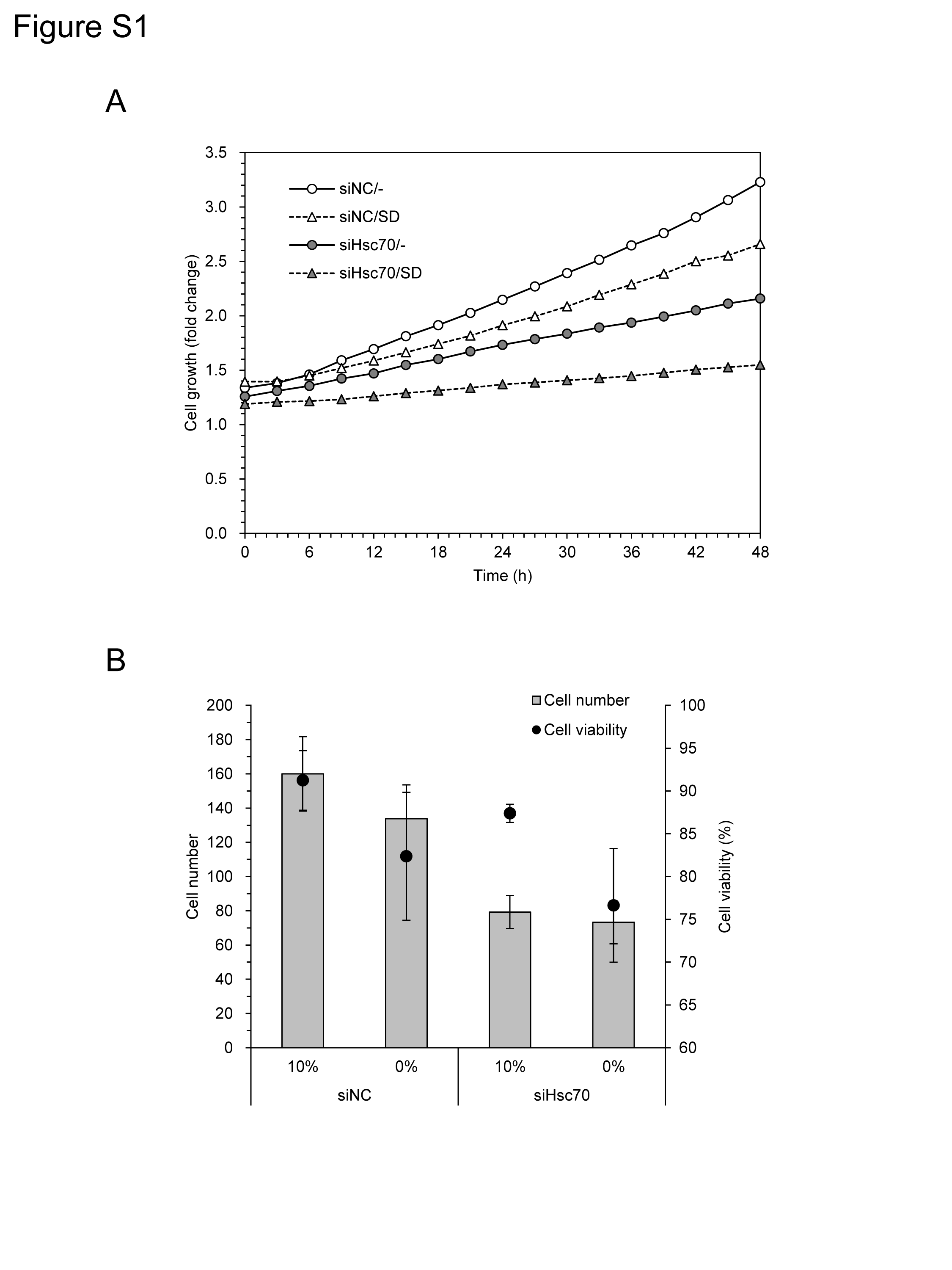

Supplement: Figure S1 — Hsc70 is critical for HT29 cell survival. (A) At 48 h after transfection of siRNA, cells were subjected to serum depletion (0% FBS) or not (10% FBS) and cell growth was monitored using IncuCyte. Images were captured every 3 h, followed by quantification of cell area in these images. (B) At 48 h after transfection, cells were subjected to serum depletion (0% FBS) or not (10% FBS) for 24 h. Both attached and detached (floating) cells were collected for trypan blue exclusion assay. Cell viability was assessed by counting trypan blue-excluding cells; values are the means ± S.D. (n = 4). (TIF) [file pone.0096785.s001.tif]

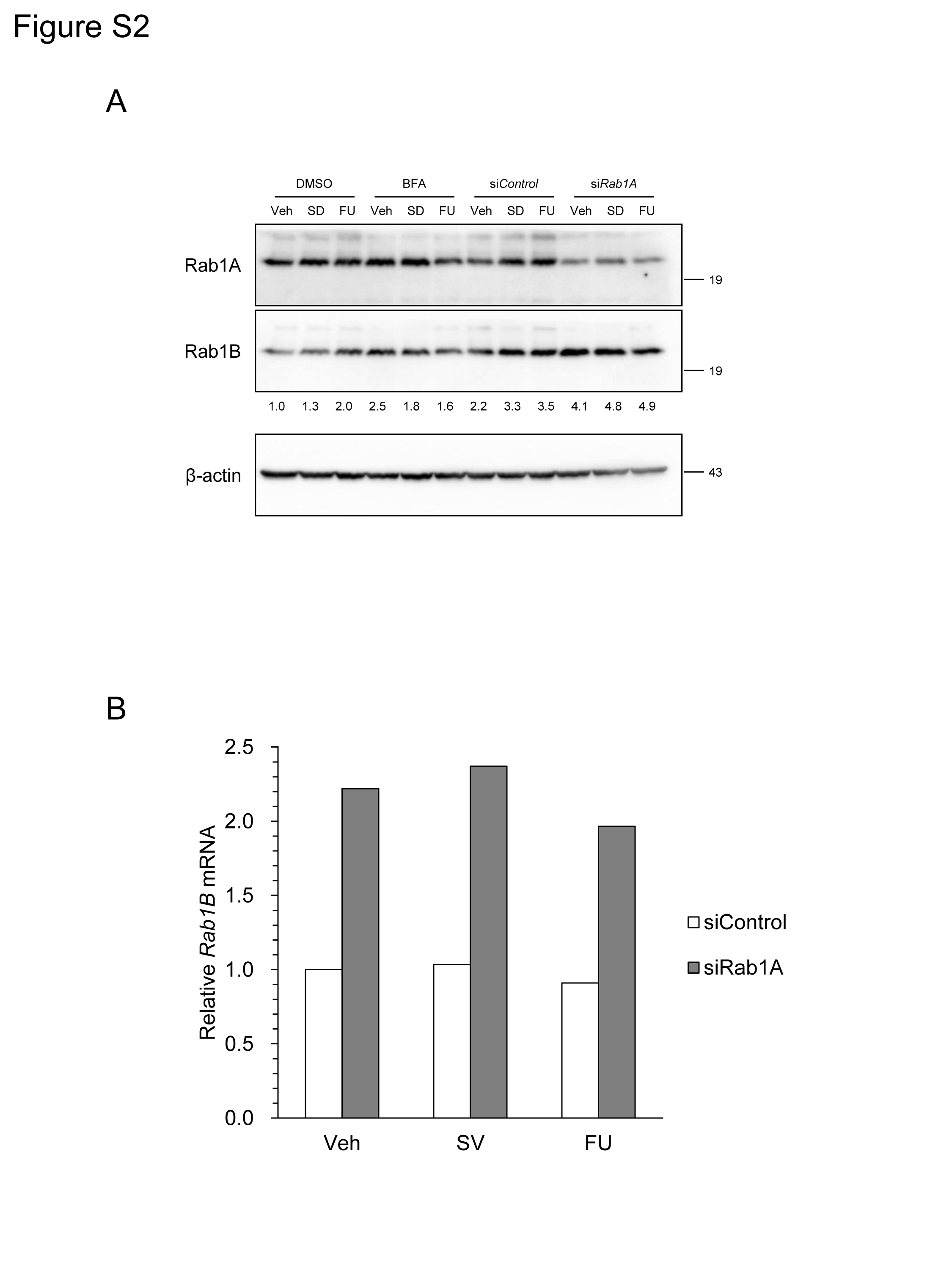

Supplement: Figure S2 — The effect of Rab1A knockdown on Rab1B expression. (A) Rab1A knockdown or control cells were subjected to serum depletion, 5-FU, or vehicle treatment for 24 h. Untransfected cells were treated with 5 µg/mL BFA for 6 h, and immunoblotted with Rab1A or Rab1B. β-actin was used as a loading control. (B) After knockdown of Rab1A, Rab1B mRNA levels were determined by qPCR at 48 h post-transfection. (TIF) [file pone.0096785.s002.tif]
